# Meta-synthesis of qualitative research on family caregiving experiences for individuals with cognitive impairment: a systematic review

**DOI:** 10.3389/fpubh.2025.1701561

**Published:** 2026-01-07

**Authors:** Yuanting Deng, Hejia Wan, Xiaohui Liu, Zilin Zhao, Xin Wang

**Affiliations:** 1 School of Nursing (Nursing School of Smart Healthcare Industry), Henan University of Chinese Medicine, Zhengzhou, China; 2 Henan Provincial Hospital of Traditional Chinese Medicine (The Second Affiliated Hospital of Henan University of Traditional Chinese Medicine), Zhengzhou, China

**Keywords:** cognitive impairment, experience, Health Care, meta-integration, qualitative research

## Abstract

**Objective:**

Qualitative studies on the caregiving experiences of families with individuals who have cognitive impairments are diverse; however, individual studies often fail to capture the multifaceted nature of caregivers' experiences. This research employed meta-synthesis to consolidate relevant findings, aiming to generate more comprehensive conclusions that can inform the development of caregiving intervention programs through systematic evidence synthesis.

**Methods:**

We conducted a computerized systematic search of the PubMed, Cochrane Library, Web of Science, ProQuest, Embase, and Scopus databases to identify qualitative studies on the caregiving experiences of family caregivers of patients with cognitive impairment, covering the period from database inception to January 2025. Study quality was assessed using the Joanna Briggs Institute Center for Evidence-Based Health Care Quality Assessment Criteria (2016). The pooled meta-integration approach was employed to reanalyze, interpret, and synthesize qualitative findings, creating new categories through systematic reclassification and aggregation.

**Results:**

A total of 15 studies met the inclusion criteria, yielding 74 distinct findings. These findings were consolidated into 15 categories representing four synthesized outcomes: (1) insufficient understanding among patients and caregivers regarding dementia etiology and symptomatology, (2) negative caregiving experiences, (3) relational challenges within familial and social networks, and (4) adaptive modifications in coping strategies for dementia.

**Conclusion:**

Family caregivers face multifaceted challenges in managing individuals with cognitive impairment; however, psychosocial support from familial, social, and societal networks facilitates positive adaptation. National governments and healthcare systems should prioritize service improvements that address the identified needs of both patients and caregivers through enhanced health service provision and targeted policy interventions.

**Systematic review registration:**

DOI: 10.17605/OSF.IO/3CZ26.

## 1 Introduction

Aging presents a universal challenge for the international community (1). As the older population rapidly expands, there is an increasing need to address the potential long-term care requirements of older adults with undiagnosed cognitive impairments (2). According to data from the World Health Organization (WHO), the global number of individuals with dementia is projected to rise from 55 million in 2019 to 139 million by 2050 (3).

Cognitive impairment refers to abnormalities in the brain's higher-order intellectual processing functions, including learning, memory, and thought processes (4). This leads to significant deficits in learning and memory and is often accompanied by pathological manifestations such as aphasia, apraxia, agnosia, or abulia. Notably, dementia represents a more profound and enduring form of cognitive impairment (5). Dementia significantly impairs an individual's capacity for academic learning, occupational performance, social engagement, and daily living activities (6). Consequently, this situation imposes significant caregiving burdens and societal strains within both familial and community contexts. From physiological and psychological perspectives, daily caregiving and sudden symptom responses contribute to poor sleep quality and increased strain. In addition, patients' health conditions and caregivers' self-neglect can lead to heightened levels of anxiety and depression (7). Socially, the loss of social interactions, imbalance between work and family, and potential prejudice exacerbate feelings of isolation (8). Furthermore, the burdens of caregiving evolve over time as the disease progresses. Given the rapid pace of population aging, there has been an annual increase in the prevalence of neurodegenerative diseases resulting from age-related cognitive dysfunction, with dementia, Alzheimer's disease, and Parkinson's disease being the most prominent (9). Cognitive disorders have thus emerged as a pressing global public health challenge, underscoring the need for enhanced long-term care services and the management of growing health-related concerns.

Influenced by the culture of filial piety, most older individuals prefer family-based caregiving, wherein informal care is provided by children, spouses, or relatives (10). These caregivers often lack formal training and possess insufficient knowledge and skills, resulting in a limited capacity for caregiving. Prolonged caregiving is linked to a cumulative physical and psychological burden, in addition to an elevated risk of cardiovascular diseases (11).

The biopsychosocial Model transcends traditional, disease-focused biomedical approaches by integrating physiological, psychological, and social dimensions to comprehensively address health and care issues (12). It directs the exploration of caregivers' multidimensional experiences—connecting physical health decline to impaired functioning, emotional distress to perceptions of cognitive decline, and role conflicts to challenges within family and social relationships (13). This model clarifies potential interactions among factors, such as physical exhaustion exacerbating emotional distress and straining family bonds, inadequate social support amplifying caregiving burdens (14), and adaptive coping necessitating multidimensional interventions. Furthermore, it informs a tripartite support system that optimizes medical resources and provides caregiver training for physiological needs, offers support groups and reduces disease-related stigma for psychological needs, and develops community programs while clarifying care responsibilities for social needs (15). Rooted in this framework, the biopsychosocial model emphasizes both patients' pathological treatment and caregivers' supportive needs while considering social environmental influences, as highlighted in relevant studies (16). This makes the recognition of caregiver burdens and needs, along with the implementation of corresponding interventions, an increasingly critical issue in geriatric health.

The number of qualitative studies focused on the caregiving experiences of caregivers for patients with cognitive impairment has been steadily increasing within the international community (17–19). However, these studies often exhibit a certain level of heterogeneity in their findings, which limits their ability to fully capture the breadth of family-based caregiving experiences. This research employed a meta-aggregation approach to consolidate qualitative research on the subjective caregiving experiences of family caregivers for patients with cognitive impairment. Through a systematic interpretation of their needs and perceptions, this synthesis aimed to provide a reference for the development of targeted interventions for caregivers. The ultimate goal was to help family caregivers perform their caregiving roles more effectively and improve their quality of life.

## 2 Materials and methods

### 2.1 Data analysis

This qualitative meta-synthesis aimed to explore the multidimensional experiences and adaptive strategies of family caregivers caring for cognitively impaired individuals. Guided by the PEO framework (20), it addressed the research question: what multidimensional experiences do these caregivers encounter? Data synthesis employed the JBI meta-aggregation approach, grounded in philosophical thinking and qualitative research methodology. Researchers systematically organized, summarized, and restructured similar study findings into new categories. They integrated the results through iterative reading, comprehension, dissection, and comparison. A total of two independent reviewers employed the Joanna Briggs Institute Qualitative Assessment and Review Instrument (JBI-QARI) (17) framework to conduct a critical appraisal of quality assessment.

### 2.2 Literature search strategy

A comprehensive search of relevant electronic databases was conducted. The databases searched included PubMed, the Cochrane Library, the Web of Science, ProQuest, Embase, and Scopus, with no restrictions on publication year, covering the period from database inception to January 2025. Qualitative studies were retrieved by combining subject headings and free words. The PICoS search strategy was established as follows: #1 “cognitive impairment”[“dementia” OR “Alzheimer disease” OR “mild cognitive impairment” OR “cognitive impairment”]; #2 ”caregiver^*^” [“spouse caregiver” OR “family caregiver^*^” OR “family” OR “informal caregiver”]; #3 “qualitative” [“Qualitative research” OR “Grounded theory” OR “Phenomenology” OR “Focus group method”]; #4 “experience^*^” [“caregiving experience” OR “care burden” OR “caregiving emotion” OR “caregiving stress”]. #5:#1 AND #2 AND #3 AND #4.

### 2.3 Characteristics of the reviewed studies

**Figure 1:**
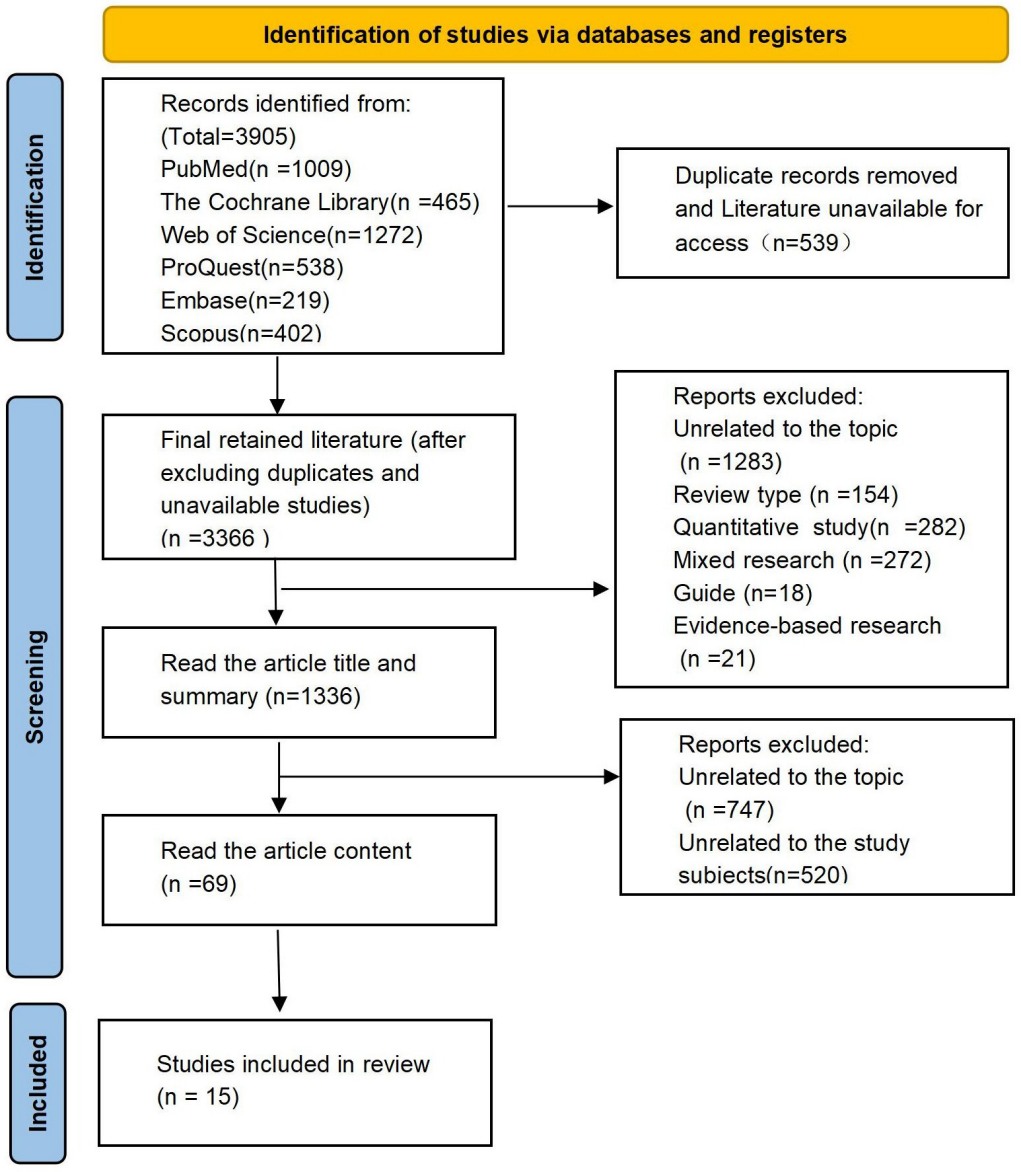
Literature retrieval and screening process.

A total of 1,336 titles and abstracts were screened, and 69 full-text articles were assessed for eligibility, resulting in the inclusion of 15 phenomenological studies (Figure 1). The studies spanned 10 countries: the United States (16, 20, 21), Canada (22), the United Kingdom (23), Lithuania (24), Sweden (25), Indonesia (18), Iran (25), Vietnam (26, 27), San Diego (28), Australia (17), and China (29), with the United States and Vietnam representing the largest proportions. The sample size of caregivers in each study ranged from 10 to 31, yielding a total of 282 participants. The majority of caregivers were either spouses or adult children of individuals with cognitive impairments, such as dementia, Alzheimer's disease, and Parkinson's disease. The research contexts included family, community, and hospital settings, with the family setting identified as the primary caregiving environment. The basic characteristics and quality evaluation results of the included literature are shown in Tables 1, 2.

**Table 1:** The basic characteristics of the included literature (*n* = 15).

**Included literature**	**Date**	**Country/Region**	**Research method**	**Research participant**	**Phenomenon of interest**	**Main results**
Owokuhaisa, J (18)	2023	United States	Phenomenological study; In-depth interviews	27 informal caregivers of dementia patients	Exploring caregiver burdens and coping strategies among caregivers of patients with cognitive impairment in rural southwestern Uganda	Eight themes: (1) Economic burden, (2) Physical and mental burden, (3) Social burden, (4) Religious coping, (5) Seeking social and emotional support, (6) Emotion-centered coping (7), Problem-centered coping, and (8) Positive coping
Ramos, M. D (19)	2023	United States	Phenomenology; In-depth interviews	15 caregivers of dementia patients	Exploring the experiences of Black women in the United States caring for family members with dementia	Five themes: (1) Family care obligations, (2) The care journey, (3) Prioritizing health issues, (4) Coping behaviors, and (5) Support needs and challenges
Janice, D. P (29)	2023	Australia	Phenomenology; Semi-structured interviews	30 caregivers of dementia patients	Exploring caregivers' perceptions of their own needs and the needs of care recipients to improve support services for dementia patients	Five themes: (1) Caregiver health loss, (2) Loss and grief, (3) Multiple care roles, (4) Family support, and (5) Economic burden
Tambunan, E. H (23)	2023	Indonesia	Interpretive phenomenology; Open-ended interviews	13 caregivers of dementia patients	Exploring the life experiences of family caregivers caring for family members with dementia	Two main themes: (1) From refusal to acceptance of care and (2) From lack of patience to empathy
Duplantier, S. C (24)	2023	United States	Phenomenology; Semi-structured interviews	10 family caregivers	Exploring barriers and facilitators to caregivers' health and wellbeing in the context of dementia support services	Five themes: (1) Time alone and the ability to prioritize oneself; (2) Positive emotional state, mental health, and social participation; (3) The subjective burden of care as a responsibility that is difficult to shake off; (4) Anticipated grief and accumulated loss; and (5) Regret and guilt, isolation and loneliness, and lack of agency
Chen MC (26)	2021	Taiwan	Phenomenology; Semi-structured, Face-to-face interviews	20 family caregivers of persons with dementia	Exploring the experiences of family caregivers and analyzing their care needs	Five themes:(1) Deciding to seek medical attention, (2) The moment of disclosure, (3) Conveying information, (4) Maintaining personal functioning, and (5) receiving support and living well with dementia
Read, J (34)	2023	United Kingdom	Phenomenology; Semi-structured interviews	11 family caregivers of patients with late-stage Parkinson's disease	Exploring the life experiences of family caregivers and their perceptions and satisfaction with service provision	Five themes: (1) Ensuring continuous support is crucial for providing care at home, (2) Utilizing multiple support sources to manage life with Parkinson's disease in the family, (3) Seeking information on proper care, (4) Perceived unmet service provision needs, and (5) Fragmentation of care for complex diseases
Kontrimiene, A (28)	2023	Lithuania	Phenomenology; Semi-structured focus groups	31 informal caregivers of older individuals with dementia	Exploring caregivers' personal experiences to improve the quality of care provided by informal caregivers	Four themes: (1) Learning care through personal experience, (2) The impact of care on social wellbeing, (3) Caregivers' conflicting emotions toward care services, and (4) Coping with challenges related to care provision
Nguyen, H (27)	2023	Vietnam	Phenomenology; Semi-structured interviews	21 caregivers of dementia patients	Understanding family caregivers' experiences of family care	Eight themes: (1) Viewing dementia symptoms as a normal part of aging rather than a disease, (2) Caregiving as a moral and expected family obligation, (3) Caregiving patterns significantly influenced by gender and sibling order, (4) Time constraints, (5) Loss of income, (6) Increased social isolation, (7) Perceived harm to their physical health, and (8) Emotional distress
Ashrafizadeh, H (25)	2023	Iran	Phenomenology; Semi-structured interviews	11 caregivers of dementia patients	Understanding the experiences of family caregivers of patients with Alzheimer's disease	Six themes: (1) Burnout and fatigue, (2) Role conflict, (3) Delegating care responsibilities to family members, (4) Neglecting the patient, (5) The economic burden of the disease, and (6) Growth and excellence
Mayo, A. M. (22)	2023	United States	Phenomenology; Semi-structured interviews	11 caregivers of dementia patients	Understanding the life experiences of caregivers of dementia patients	Five themes: (1) Disease uncertainty, (2) Loneliness, (3) The complexity of frustration, (4) Positive caregiving experiences, and (5) Gratitude
Nguyen TTT (21)	2025	Vietnam	Phenomenology; Semi-structured interviews	20 caregivers of dementia patients	Exploring the challenges faced by family caregivers in providing care for people with dementia	Six themes: (1) Challenges in daily care, (2) Strain from behavioral and sleep disruptions, (3) Difficulties in seeking help, (4) Personal sacrifices of caregivers, (5) Emotional burdens, and (6) Limited dementia knowledge and caregiving skills
Lethin, C (35)	2016	Sweden	Phenomenology; Focus group interviews	23 family caregivers of patients with late-stage Parkinson's disease	Investigating family caregivers' formal care experiences while caring for dementia patients	Three themes: (1) Care stress and fatigue, (2) Feeling lonely and isolated, and (3) Need for preparation and knowledge about dementia disease
Peacock, S. C (36)	2014	Canada	Phenomenology; Open-ended interviews	11 caregivers of dementia patients	Exploring the dementia care process for family caregivers who have lost a loved one	Three themes: (1) Receiving the diagnosis, (2) Managing at home (home care and finding strength), and (3) Transitioning to long-term care
Teti, M (37)	2023	United States	Phenomenology; Semi-structured interviews	28 family caregivers of dementia patients	Exploring the daily care experiences and needs of family caregivers of dementia patients	Three themes: (1) Behavioral changes, (2) Managing dual roles, and (3) Recognizing the severity of the disease

**Table 2:** Critical appraisal of the included studies (*n* = 15).

	**The JBI-QARI**											**Dependability**
**Included study**	**Q1**	**Q2**	**Q3**	**Q4**	**Q5**	**Q6**	**Q7**	**Q8**	**Q9**	**Q10**	**Score**	
Owokuhaisa, J (18)	Unclear	Yes	Yes	Yes	Yes	No	No	Yes	Yes	Yes	7/10	Moderate
Ramos, M. D (19)	Yes	Yes	Yes	Yes	Yes	No	No	Yes	Yes	Yes	8/10	Moderate
Janice, D. P (29)	Unclear	Yes	Yes	Yes	Yes	No	No	Yes	Yes	Yes	7/10	Moderate
Tambunan, E.H (23)	Unclear	Yes	Yes	Yes	Yes	No	No	Yes	Yes	Yes	7/10	Moderate
Duplantier, S.C (24)	Yes	Yes	Yes	Yes	Yes	No	No	Yes	Yes	Yes	7/10	Moderate
Chen MC (26)	Yes	Yes	Yes	Yes	Yes	No	No	Yes	Yes	Yes	8/10	Moderate
Read, J (34)	Yes	Yes	Yes	Yes	Yes	No	No	Yes	Yes	Yes	8/10	Moderate
Kontrimiene, A (28)	Yes	Yes	Yes	Yes	Yes	No	No	Yes	Yes	Yes	8/10	Moderate
Nguyen, H (27)	Yes	Yes	Yes	Yes	Yes	No	No	Yes	Yes	Yes	8/10	Moderate
Ashrafizadeh, H (25)	Yes	Yes	Yes	Yes	Yes	No	No	Yes	Yes	Yes	8/10	Moderate
Mayo, A.M (22)	Unclear	Yes	Yes	Yes	Yes	No	No	Yes	Yes	Yes	7/10	Moderate
Nguyen TTT (21)	Yes	Yes	Yes	Yes	Yes	No	No	Yes	Yes	Yes	8/10	Moderate
Lethin, C (35)	Yes	Yes	Yes	Yes	Yes	No	No	Yes	Yes	Yes	8/10	Moderate
Peacock, S.C (36)	Yes	Yes	Yes	Yes	Yes	No	No	Yes	Yes	Yes	8/10	Moderate
Teti, M (37)	Yes	Yes	Yes	Yes	Yes	No	No	Yes	Yes	Yes	8/10	Moderate

Whether the philosophical foundation and methodology are consistent; Whether the methodology is consistent with the research question or objective; Whether the methodology is consistent with data collection methods; Whether research methods are consistent with data analysis and presentation methods; Whether the methodology is consistent with the interpretation of results; Whether the researcher's concepts and values that may influence the research have been clarified; Whether the researcher's influence on the research and the research's influence on the researcher have been explained; Whether the participants' stated intentions are adequately represented; Whether it complies with current ethical standards; and Whether the research conclusions are derived from the analysis and interpretation of the data.

JBI-QARI, Joanna Briggs Institute Qualitative Assessment and Review Instrument.

According to the JBI-QARI, the 15 included phenomenological studies exhibited moderate overall quality, with scores ranging from 7 to 8 out of 10. All four synthesized findings received moderate ConQual scores. Dependability was uniformly downgraded due to methodological limitations. In contrast, credibility was reduced for Findings 1, 3, and 4 due to issues related to sample size, data quality, and cultural gaps, while Finding 2 maintained its credibility due to comprehensive burden verification (see Table 3).

**Table 3:** ConQual summary of the findings.

**Synthesized finding**	**Design**	**Dependability**	**Credibility**	**ConQual score**	**Comments**
Synthesized finding 1: lack of understanding regarding the etiology and symptoms of cognitive impairment among both patients and their caregivers. This includes notable gaps in knowledge concerning disease progression, the psychological changes experienced by patients, and the distress caused by various symptoms	Qualitative	Downgraded by 1 level: initial inter-rater agreement for literature screening and assessment was 85%; and the included studies were of moderate quality (7/10 points)	Downgraded by 1 level: samples included 282 caregivers (10 countries/multiple scenarios) but were geographically concentrated (US/Vietnam); no gray literature was included, introducing publication bias	Moderate	Clarified knowledge gaps, but generalizability was limited by sample size and bias
Synthesized finding 2: negative caregiving experiences encompass various burdens, including physical, psychological, and financial strains, as well as unmet help-seeking needs	Qualitative	Downgraded by 1 level: dual extraction ensured consistency; the included studies were of moderate quality, and some studies did not clarify data saturation criteria	No change	Moderate	Comprehensively presented negative experiences, but relevance was limited by sample size and disease types
Synthesized finding 3: familial and social relationship challenges arise, characterized by difficulties in role adaptation, limited social interaction, and family conflicts	Qualitative	Downgraded by 1 level: dual coding and third-party review were applied, but some studies provided insufficient interpretation of cultural backgrounds	Downgraded by 1 level: studies covered core relationship issues (including cultural factors) but provided a vague description of deficiencies in community support	Moderate	Defined dimensions of relationship challenges, but depth/timeliness was limited by cultural interpretation and design
Synthesized finding 4: caregivers often employ adaptive coping strategies, such as adjusting their self-perception, acquiring relevant knowledge, and reconstructing family relationships	Qualitative	Downgraded by 1 level: subjective bias was present in strategy extraction; only three studies mentioned digital tools, with weak evidence strength	Downgraded by 1 level: studies covered diverse coping pathways but did not differentiate cultural differences and lacked follow-up data on strategy effectiveness	Moderate	Systematically organized strategies, but generalizability was limited by bias and evidence

### 2.4 Literature inclusion and exclusion criteria

The inclusion criteria were as follows: (1) participants: formal family caregivers of cognitively impaired patients, both domestically and internationally; (2) phenomenon of interest: psychological experiences, feelings, and needs of caregivers of cognitively impaired patients; (3) study settings: Hospitals, communities, and families; and (4) types of studies: qualitative research, including phenomenological, grounded theory, ethnographic, narrative, and descriptive studies.

The exclusion criteria were as follows: (1) studies that do not provide significant insights into family caregiver experiences; (2) studies focusing on the experiences of individuals with cognitive impairments or healthcare professionals; (3) literature for which full-text access is not available; (4) duplicate publications: studies from the same research team using identical data, participants, and conclusions, regardless of the publication venue, to avoid data redundancy and biased synthesis; and (5) non-qualitative studies, including mixed-methods studies, quantitative studies, reviews, and guideline reports. This meta-synthesis specifically focused on the qualitative exploration of subjective caregiving experiences, as the aforementioned study types do not contain relevant original qualitative data or cannot be aligned with the synthesis methodology.

### 2.5 Literature screening

All retrieved records were imported into NoteExpress to remove duplicate studies. A total of two reviewers independently screened all titles and abstracts obtained from the searches; full manuscripts of potentially relevant studies were retrieved and assessed by the two reviewers in accordance with the inclusion criteria. The initial inter-rater agreement for both literature screening and study evaluation was 85%. Any discrepancies arising during these processes were resolved through adjudication by a third investigator, with final consensus reaching 100% following resolution.

### 2.6 Data extraction

A standardized data extraction form was designed to facilitate the electronic comparison of the study entries. In addition, two researchers independently extracted data from the included studies using this form, ensuring that the extracted information encompassed the author(s), publication year, country, study population, methodology, phenomenon of interest, and key findings. For the extraction of key findings, a clear criterion was established: if a study reported multiple sets of results pertaining to the same core theme, only those directly addressing the caregiving experiences of family caregivers were considered.

### 2.7 Quality assessment

A total of two independent researchers assessed the methodological quality of each eligible study using the JBI-QARI. The tool comprises 10 items, including research methodologies, philosophical foundations, research purposes, data collection methods, data analysis methods, consistency in the interpretation of results, source of conclusions, consideration of researcher influence on the study, representativeness of participants, and ethical considerations. For each item, responses were categorized as “yes” (if the criteria were met), “no” (if the criteria were not met), “unclear” (if it was uncertain whether the criteria were met), or “not applicable” (if the criteria were not applicable). Any discrepancies between the two researchers during the assessment were resolved through structured discussion. To avoid missing potentially valuable data, this review did not exclude articles based on their quality scores.

## 3 Results

**Figure 2:**
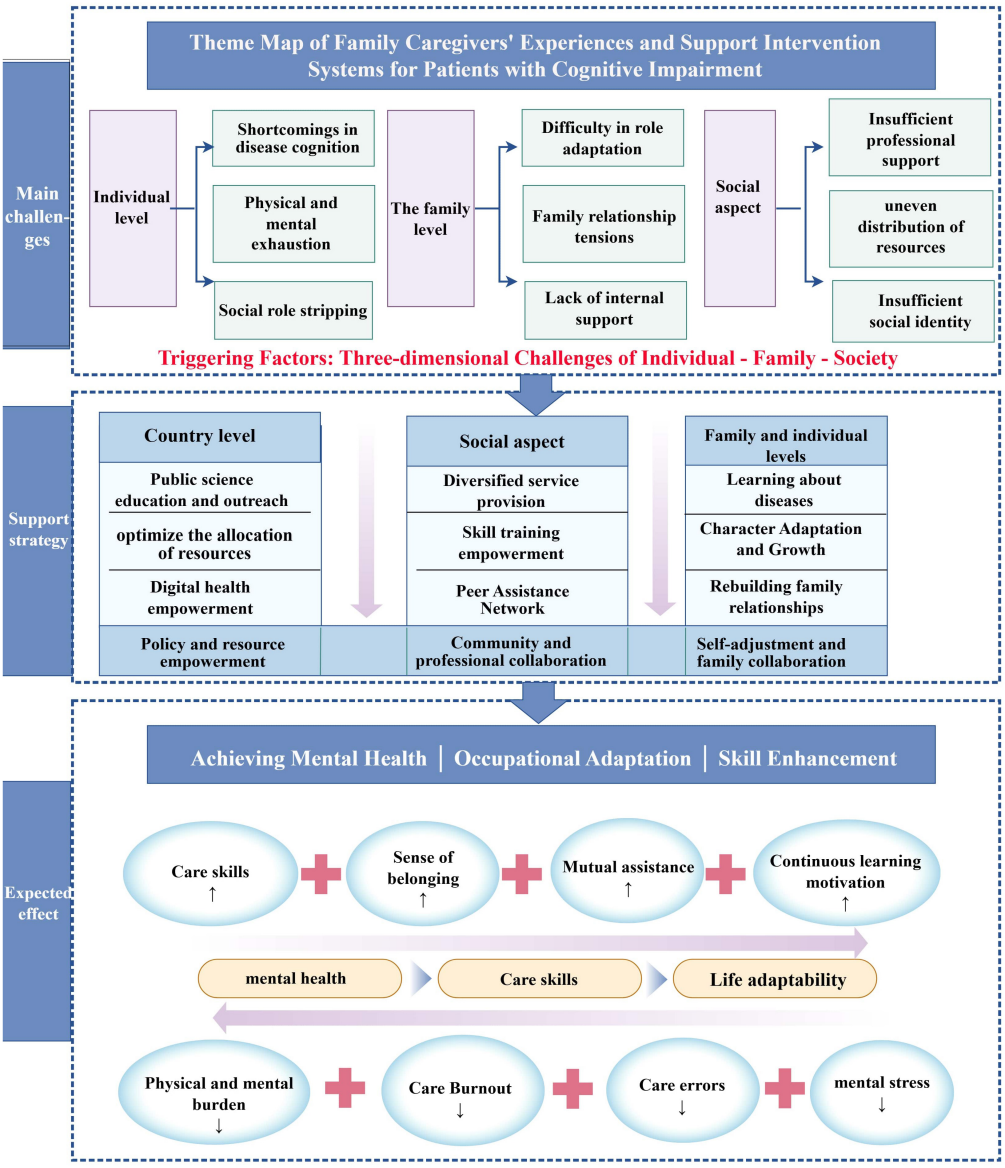
Theme map of family caregivers' experiences and support intervention systems for patients with cognitive impairment.

The researcher repeatedly reviewed and analyzed the included literature, distilling 74 clear findings. Similar findings were summarized and grouped into 15 new categories, which were further synthesized to obtain four integration results: caregivers'/patients' lack of understanding of the causes and symptoms of dementia, caregivers' negative experiences, challenges in family and social relationships, and adaptive changes to cope with the situation. Figure 2 illustrates the “Theme Map of Family Caregivers' Experiences and Support Intervention Systems for Patients with Cognitive Impairment.”

### 3.1 Integration outcome 1: self-perception of cognitive decline

#### Category 1: lack of knowledge

Cognitive impairment progresses gradually, and caregivers—mostly domestic partners—lack knowledge about disease development. This contributes to high levels of uncertainty regarding the condition (“Unless you've already taken care of someone, you really don't know what kind of care to provide”) (13) and the patient's future prognosis (“I doubt that I will be able to care for him”) (18).

#### Category 2: complex psychological changes

Patients experiencing high levels of disease-related stigma may show more stressful reactions (“She gets angry now and then... It's very frustrating. It makes me anxious.”) (9). Some patients develop feelings of incompetence and low self-esteem and reject or avoid social contact due to behavioral changes associated with impaired self-perception (“When we go out, we sit where people can't see us”) (29).

#### Category 3: symptom distress

Patients with cognitive impairment experience a gradual decline in emotional regulation, memory, and verbal expression with age and the progression of the illness. This may manifest as emotional irritability (“lashes out for no apparent reason and sometimes cries”) (11), forgetfulness of recent or distant events (“Tell him something and forget it in the twinkling of an eye”), and repetitive speech (“He always repeats what he is told”) (18). [sic].

### Integration outcome 2: negative caregiver experiences

#### Category 1: impaired physical functioning

Family caregivers of individuals with cognitive impairment frequently report negative experiences related to caregiving, which are often characterized by profound physical fatigue. As one caregiver noted, (“When I work, I feel very tired”) (6). Furthermore, the dual burdens of caregiving, which include daily care responsibilities and the need to adapt to the patient's condition, significantly exacerbate both physical and mental exhaustion among caregivers. As another caregiver expressed, (“I feel really tired when I'm there”) (13). Some caregivers may develop negative emotions such as burnout, depression, or frustration as a result of long-term caregiving (“I unloaded my emotions and the doctor told me I could let them out”) (13).

#### Category 2: psychological maladjustment

Caregivers of people with dementia are often physically and mentally constrained in their caregiving roles (“like a prisoner; in fact, a hostage to my mother”) (15), experiencing fear, anxiety, and emotional distress (“I can't tolerate his behavior and get angry or suspicious”) (13).

#### Category 3: financial and treatment stress

Although conventional medication can be effective, it is not a short-term solution and often imposes substantial financial costs and side effects (“Caring for him must cost 500,000–700,000 Tomans per month for medication”) (15). In addition, when caregivers themselves require hospitalization, this creates further therapeutic and financial burdens (“I just had cervical spine surgery myself, but if I don't take care of her, she will starve to death”) (20).

#### Category 4: negative experiences of help-seeking for caregivers

Caregivers are often disappointed that they do not receive the family support they expect, leading to increased feelings of isolation (“We used to have a lot of friends. They've disappeared......”) (17). They also want to seek help from professionals to obtain formal caregiving knowledge but become discouraged when they are not given adequate guidance (“My husband had a stroke and he couldn't stand up. After 2 days, the nurse sent him back from the hospital without giving any care plan”) (19).

### 3.3 Integration outcome 3: challenges in family and social relationships

#### Category 1: family role maladjustment (caregiver role deficit and enhancement)

It is initially difficult for family caregivers to accept the caregiving pressures that arise from the patient's loss of self-care (“I don't accept my father's condition because he is old and just retired”) (9). Role overload also occurs when work and caregiving responsibilities conflict (“He even had to take unpaid leave because we had to take care of our father”) (9).

#### Category 2: changed life status

Caregivers often struggle to balance their work life and caregiving responsibilities and feel stuck (“As a family caregiver, I not only take care of my family, but I also have to take time to enjoy life”) (20). In addition, their engagement in other social roles becomes increasingly limited, and their overall life status changes (“I don't go anywhere except to work because I don't feel comfortable”) (6).

#### Category 3: loss of self-concept

Most family caregivers perceive the burden of the caregiving role as an inescapable responsibility, which often affects their own sense of initiative (“I feel lost... But I don't know what to do”) (10). Over time, they become trapped in the caregiving role and begin to lose their self-concept (caregiver, mom...) (“Along the way I gradually lost myself.”) (19).

### 3.4 Integration outcome 4: making changes to respond positively

#### Category 1: self-awareness and role adaptation

Caring for a family member with cognitive impairment provides caregivers with life experience and a sense of accomplishment that motivates them to continue in their caregiving role and strengthens their sense of self-worth (“I learned the value of patience and respect for my parents from my experience caring for my parent with Alzheimer's disease.”) (9). [sic].

#### Category 2: personal growth and enhanced resilience

Caregivers' demanding experiences shape the development of positive personality traits (“At first, I didn't think I could do it... but I went looking and researched and I did it, with a lot of support from my family”) (29). By adapting positively and responding effectively, caregivers are able to maintain a relatively stable physical and mental state, which enhances their resilience (“I started researching the disease and I feel like I've matured”) (9). [sic].

#### Category 3: seeking knowledge of the disease from multiple sources

Caregivers are proactive in understanding the type and progression of cognitive impairment and in learning how to meet the changing needs of the person they care for throughout dementia care (“I'm coming to understand dementia.... I think I did”) (14). Some family caregivers also gain knowledge and experience in dementia care through peer-to-peer exchanges or online platforms (“I communicated with friends and received information about how to deal with my father's changing behavior and encouraged me”) (10). [sic].

#### Category 4: gaining patient support and rebuilding family relationships

Caregivers gain support from the patient and family, which enhances their sense of fulfillment and wellbeing regarding the patient's recovery and peace of mind [“I've been taking care of him for almost 10 years now and it's just like a family member” (18)]. They also use companionship as a link between family caregiving and maintaining daily life (“I feel happy when I see that he/she is better because of my care”) (16).

## 4 Discussion

This study systematically summarizes the multidimensional experiences of family caregivers for individuals with cognitive impairments through meta-integration, identifying four major findings: (1) insufficient understanding of the disease by both caregivers and patients, (2) negative experiences encountered by caregivers, (3) challenges in familial and social relationships, and (4) adjustments made to foster positive coping strategies. The subsequent discussion is organized across individual, family, social, and national levels, offering targeted recommendations.

### 4.1 Insufficient disease cognition as the primary bottleneck affecting care quality

This study found that caregivers generally exhibit a serious lack of understanding regarding the causes, progression, and prognosis of cognitive impairments (18). This knowledge gap leads to biases in caregiving decisions, such as attributing abnormal behaviors of patients to “normal aging” or avoiding professional intervention due to traditional beliefs (13). Some caregivers rely solely on limited caregiving experiences, lacking a systematic understanding of the disease's progression, which further exacerbates the blind nature of caregiving and leads to ineffective coping. Therefore, enhancing caregivers' understanding of the nature and trajectory of the disease is fundamental to improving caregiving practices.

### 4.2 Negative caregiving experiences exhibit multidimensional and overlapping characteristics

Caregivers bear significant burdens across physiological, psychological, economic, and social dimensions. Physiologically, long-term caregiving leads to physical exhaustion and health deterioration; psychologically, caregivers experience anxiety, depression, and emotional breakdowns (16); economically, medical costs and caregiving-related expenses create continuous pressure; and socially, being forced to withdraw from social networks due to caregiving responsibilities deepens feelings of isolation. These burdens are particularly pronounced among caregivers in culturally specific contexts or geographically remote areas. Therefore, local beliefs and values should be considered when designing caregiving support systems. The expansion of telemedicine and mobile clinic services should be prioritized to address gaps in caregiving support systems and to alleviate social isolation among caregivers.

### 4.3 Challenges in restructuring family and social relationships

During the caregiving process, overloaded family roles, conflicts of responsibility, and a lack of social support are common issues (16). Some caregivers experience dual-role pressure from balancing cultural heritage preservation with caregiving duties (18). Tensions often arise among family members due to disputes over the allocation of caregiving responsibilities and communication barriers. In addition, inadequate professional support systems exacerbate caregivers' feelings of helplessness. Therefore, establishing a social support network that provides emotional support, professional guidance, and resource coordination is critical for mitigating interpersonal stress.

### 4.4 Active adaptation depends on three-dimensional synergy across the individual, family, and society

Despite numerous challenges, some caregivers achieve positive coping and personal growth through role adaptation, proactive learning, and the reconstruction of family support (10). They enhance caregiving resilience by acquiring disease knowledge through multiple channels, participating in community mutual aid programs, and adjusting their mindset and expectations (14). Cultural identification and shared responsibilities within families provide sustained motivation, while professional support and resource integration at the social level serve as external safeguards. This suggests that caregiver adaptation is a dynamic process involving multi-level interactions, highlighting the need for an empowering framework that integrates individual capabilities and family support.

### 4.5 Building a tripartite support system involving the state, society, and family

#### National level: expand and integrate mobile health services

The government, in collaboration with media and public welfare organizations, should implement cognitive impairment-focused science communication initiatives—including short videos, educational pamphlets, and community lectures—with nursing personnel responsible for designing specialized content (30). Simultaneously, efforts should be made to optimize the allocation of dementia care resources and to enhance professional training programs. In addition, governments should prioritize the expansion of Internet-based and electronic health services, especially in remote and rural areas. By leveraging digital technologies for intelligent health education and professional caregiver training (31), these initiatives can alleviate family caregivers' burden, improve healthcare efficiency, and potentially reduce overall medical costs.

#### Social level: consolidate resources to develop diverse community–family care programs

A multi-faceted care system should be established by utilizing information platforms such as telephone follow-ups, thematic lectures, home visits, and social media (32). Healthcare professionals should organize regular online and offline health education sessions and caregiver support groups while developing and distributing practical care manuals covering symptom recognition, daily care strategies, and communication techniques. Strengthening public awareness through community-based science communication and facilitating peer and professional support can further enhance caregivers' competence and psychological resilience.

#### Family and individual level: provide personalized and stage-appropriate support

The prevalence of cognitive impairment-based chronic diseases is increasing annually, and reliance on family or friends for caregiving support often has a negative impact on the health of caregivers. Studies have shown that Internet-based interventions have brought about positive mental health outcomes for caregivers (33). Intelligent health education and specialized caregiving skills training delivered through electronic information technologies help family caregivers manage their burdens and challenges while potentially reducing healthcare costs. Therefore, national governments should expand the availability of Internet-based and e-medicine services for family caregivers across all regions, especially in remote and rural areas, to ensure healthcare accessibility and equity. They should also take into account the cultural context of the people in the region and the background of medical facilities, integrating mHealth into hospitals, communities, families, and patients as a tool to strengthen the continuity of care. Efforts should focus on improving the care model for delivering mHealth services and developing a holistic mHealth care service system that connects hospitals, communities, and families.

## 5 Conclusion

This study systematically synthesizes the multidimensional caregiving experiences of family caregivers for individuals with cognitive impairments through a meta-synthesis approach, yielding three major conclusions. First, the research confirms that caregivers commonly face a core dilemma characterized by insufficient disease-related knowledge combined with cumulative negative experiences, providing empirical evidence for the development of inclusive caregiving policies. Second, it reveals the profound impacts of cultural adaptation and geographic isolation on caregiving experiences, highlighting the necessity of prioritizing personalized and locally adapted services in caregiving support design. Third, the study clarifies empowerment pathways for caregivers, encompassing individual adjustment, family support, and social resource integration, offering theoretical references for constructing systematic intervention models. However, this study has certain limitations. The included literature primarily consisted of cross-sectional qualitative studies, which limits the ability to draw causal inferences. In addition, samples were predominantly drawn from published studies with focused perspectives, potentially limiting the generalizability of the results. Furthermore, the search strategy did not comprehensively cover gray literature and was dominated by foreign-language publications, whose applicability in local contexts and cultural settings requires further validation. Subjective judgments during theme synthesis may also introduce heterogeneity and potential bias. Drawing from these findings and their limitations, future research should consider several avenues for expansion. First, it is imperative to broaden the scope of study populations to address the evolving needs associated with various cognitive impairments and stages of care. Second, adopting longitudinal designs will allow for tracking the progression of caregiving experiences, enabling the identification of pivotal moments for interventions.

## Data Availability

The original contributions presented in the study are included in the article/supplementary material, further inquiries can be directed to the corresponding author.
